# End of life care for long-term care residents with dementia, chronic illness and cancer: prospective staff survey

**DOI:** 10.1186/s12877-019-1159-2

**Published:** 2019-05-22

**Authors:** Michal Boyd, Rosemary Frey, Deborah Balmer, Jackie Robinson, Heather McLeod, Susan Foster, Julia Slark, Merryn Gott

**Affiliations:** 1 School of Nursing, The University of Auckland, Private Bag, Auckland, 92019 New Zealand; 2 Freemasons’ Department of Geriatric Medicine, The University of Auckland, Auckland, New Zealand

**Keywords:** Residential facilities, Palliative care, Frail elderly, End-of-life care, Quality of dying

## Abstract

**Background:**

Little is known about the quality of end of life care in long-term care (LTC) for residents with different diagnostic trajectories. The aim of this study was to compare symptoms before death in LTC for those with cancer, dementia or chronic illness.

**Methods:**

After-death prospective staff survey of resident deaths with random cluster sampling in 61 representative LTC facilities across New Zealand (3709 beds). Deaths (*n* = 286) were studied over 3 months in each facility. Standardised questionnaires - Symptom Management (SM-EOLD) and Comfort Assessment in End of life with Dementia (CAD-EOLD) - were administered to staff after the resident’s death.

**Results:**

Primary diagnoses at the time of death were dementia (49%), chronic illness (30%), cancer (17%), and dementia and cancer (4%). Residents with cancer had more community hospice involvement (30%) than those with chronic illness (12%) or dementia (5%). There was no difference in mean SM-EOLD in the last month of life by diagnosis (cancer 26.9 (8.6), dementia 26.5(8.2), chronic illness 26.9(8.6). Planned contrast analyses of individual items found people with dementia had more pain and those with cancer had less anxiety. There was no difference in mean CAD-EOLD scores in the week before death by diagnosis (total sample 33.7(SD 5.2), dementia 34.4(SD 5.2), chronic illness 33.0(SD 5.1), cancer 33.3(5.1)). Planned contrast analyses showed significantly more physical symptoms for those with dementia and chronic illness in the last month of life than those with cancer.

**Conclusions:**

Overall, symptoms in the last week and month of life did not vary by diagnosis. However, sub-group planned contrast analyses found those with dementia and chronic illness experienced more physical distress during the last weeks and months of life than those with cancer. These results highlight the complex nature of LTC end of life care that requires an integrated gerontology/palliative care approach.

## Background

It is well known that the population is ageing and this changing demographic is impacting where people die [1]. The accumulation of multiple co-morbidities as people age often results in an extended period of physical and functional decline requiring 24-h care in a long-term care (LTC) setting [2]. In resource rich countries up to a third of people over 65 years old will die in a LTC setting and of those, more than a third will die within 6 months of admission [1, 3]. Palliative care in old age is frequently complicated by an extended period of physical and cognitive decline associated with advanced frailty requiring a different approach than traditional models of palliative care which grew primarily from cancer care [4, 5]. Long Term Care (LTC) facility staff must be skilled in providing excellent care to promote quality of life for people with complex co-morbidities and geriatric syndromes, as well as advanced palliative and end of life care. They must also provide support to families who may be distressed due to anxiety and/or grief related to the physical and/or cognitive decline of their relative [6]. However, it has only been within the last few decades that the high level of palliative care needs in LTC facilities has been recognised and researched [7].

There are many barriers to high-quality palliative care for those with advanced frailty, particularly for the large majority of LTC residents who have some form of cognitive impairment [8]. People who die of or with dementia often experience severe symptoms such as pain, fear, and anxiety, as well as clinical complications such as pneumonia and other infections, incontinence, pressure injury, cachexia and dehydration [9, 10]. They also have difficulty communicating their needs and preferences, consequently increasing the risk of suffering at the end of life [11].

In recent years there has been a significant increase in research about the palliative care needs for those with dementia, and research comparing the differences in palliative care needs between those with cancer and those with dementia. However, relatively little research has been conducted comparing quality of end of life care across different diagnostic categories for older people living in LTC facilities [12, 13]. In a widely quoted model, Lynn and Adamson (2003) describe three theoretical trajectories of functional decline prior to death: 1) the typical cancer trajectory with a short period of physical decline, 2) organ system failure with intermittent exacerbations and the potential for sudden death at any time, and 3) ‘prolonged dwindling’ typical of people with dementia, stroke and frailty [14]. Although there is relatively little empirical evidence to support the reality of Lynn and Adamson’s theoretical model, it is a useful and pragmatic way to classify different end of life courses for older people living in LTC settings [15]. The focus of this study was to use these three end of life trajectory categories (referred here as cancer, dementia or chronic illness) to describe the end of life experience of those living in LTC facilities in New Zealand through an after-death study with prospective monitoring of resident deaths over 3 months.

We report the results of post-mortem surveys with facility managers, registered nurses (RNs), and general medical practitioners (GPs), or nurse practitioners (NP) most closely associated with the care of the residents who died in a representative sample of LTC facilities. This study utilised the methods of a nationwide post-mortem study of dementia deaths in LTC facilities in Belgium and responds to the identified need for international comparison studies in palliative care [10, 16].

## Methods

### Design

Staff survey of the quality of resident death through prospective monitoring of resident deaths over 3 months.

### Sample

Facilities were stratified by region, size (up to 70 beds, and over 70 beds), and by for-profit or not-for-profit status. A random cluster sample of 61 representative facilities was selected and as facilities declined to participate, another facility was recruited until each stratified category had a proportional representative sample. All resident deaths (occurring in the facility or elsewhere) during a specified three-month period were recorded by facility administration. Data collection occurred from January 2016 to February 2017. The researchers contacted facilities weekly to ascertain the number of deaths that had occurred. All resident identifying information was kept anonymous to researchers, although the facility kept a record of resident name, national health identification (NHI) number and correlating study number. For all those who died, a list of diagnoses from the facility clinical record at the time of death was obtained. Researchers facilitated questionnaire completion through on-site follow up at the facility. The questionnaires included primary demographics, level of care at the time of death, cardiopulmonary resuscitation (CPR) status, evidence of a named enduring power of attorney (proxy decision-maker), and any advanced care-planning information. After-death staff questionnaires were used to survey GPs/NP and RNs involved in the resident’s care to confirm dementia diagnosis using the Global Deterioration Scale [10]. Decedents with both cancer and dementia were placed in a separate category. Lastly, if the resident had not been classified as either in the dementia or cancer category, they were placed in the chronic illness category.

The majority of information about end of life circumstances and quality of death was obtained from the RN identified by the facility manager as primarily involved in the resident’s care in the last week of the decedents life. These RNs were asked about the resident’s condition 1 month before death and asked to identify sentinel events [pneumonia, problems eating or drinking, stoke, cancer, febrile episodes, hip fracture, gastrointestinal bleed and other) [10]. They were also asked about symptom management 1 month prior to death using the Symptom Management at End-of-Life in Dementia (SM-EOLD), such as management of restlessness, depression, fear and anxiety, pain, shortness of breath, and skin breakdown [17]. RN staff were asked if:

- the family/friends were involved in the care during the last month of life
- the resident was able to express wishes about medical decisions
- an end of life pathway/guideline was used
- staff from the local hospice organisation were involved in the care

RNs were also asked if the resident had the following issues 1 week prior to death: decubitus ulcer, urinary or bowel incontinence, restraint use, difficulties eating and hydration status. They were also asked if they judged the death as expected or unexpected. Comfort in the last week of life was determined using the Comfort Assessment at End-of-Life in Dementia (CAD-EOLD) [17]. Previous reviews have recommended these tools as appropriate to measure quality of dying for the LTC population with various degrees of cognitive and physical disability [18–20]. Ethical approval was obtained from University of Auckland Human Participants Ethics Committee (Phase One ref. 015461 and Phase Two ref. 015650).

### Statistical analysis

Descriptive statistics were used as well as differences in distribution between diagnosis category using Pearson’s Chi Square and Fisher Exact Tests (*p* < .05). Medians and averages were tested using Kruskal-Wallis and analysis of variance (p < .05) using SPSS software version 22. All analyses compared diagnoses of cancer, dementia or chronic illness; the small number of cases with dual diagnoses of cancer and dementia were analysed as a separate category. Planned contrast analyses between diagnostic groups were conducted following significant ANOVA results for differences in CAD-EOLD and SM-EOLD individual item scores. Multivariate analysis of variance was conducted to examine the effect of interactions between decedent diagnoses and hospice involvement on comfort levels as measured by CAD-EOLD subscales.

## Results

Of the 116 facilities approached, 53% (*n* = 61) agreed to be part of the research and they recorded 286 deaths over the study period (Table 1). Primary diagnoses included: cancer (17% of total sample), dementia (49%), chronic illness (30%), and both cancer and dementia (4%). More males had a diagnosis of cancer (58%) and more females (60%) had a diagnosis of dementia (× 2(2) = 7.76, *p* = .02).

**Table 1:** Facility characteristics: Frequency and Percent n(%)(*n* = 61)

		Region			
	Total	1	2	3	4
Number of facilities	61 (100)	21 (34.4)	14 (22.9)	21 (34.4)	5 (8.1)
Total number beds:	3709 (100)	1151 (31.0)	926 (24.9)	1300 (35.0)	332 (8.9)
Low level care	1669 (100)	525 (31.4)	313 (18.7)	690 (41.3)	141 (8.4)
High level care	1634 (100)	543 (33.2)	474 (29.0)	480 (29.3)	137 (8.3)
Secure dementia	406 (100)	83 (20.4)	139 (34.2)	130 (32.0)	54 (13.3)
Number of facilities by size:					
< 70 beds	44 (100)	15 (34)	9 (20.4)	16 (36.3)	4 (9.0)
> 70 beds	17 (100)	5 (29.4)	5 (29.4)	6 (35.2)	1 (5.9)
Business type:					
For profit facilities	39 (100)	15 (38.4)	9 (23.0)	14 (35.8)	1 (2.5)
Not for profit facilities	22 (100)	5 (22.7)	5 (22.7)	8 (36.3)	4 (18.1)

People with cancer were younger compared to those with dementia or chronic illness (15%) (× 2(4) = 30.53, *p* = .00) (Table 2). Length of stay ranged from less than 1 day to 18 years, with a median length of stay of 11 months (interquartile range 31). The median length of stay was 1 month for those with cancer (interquartile range 6), 15 months for those with dementia (interquartile range 33) and 17 months for those with chronic disease (interquartile range 36). A Kruskal Wallis test demonstrated significant differences in length of stay based on diagnosis (H (2) = 43.00, *p* = .00) with a mean rank of 59 for decedents with cancer, 134 for those with dementia and 136 for those with chronic illness. Those with dementia had higher rates of faecal incontinence (79%) compared to those with chronic illness (65%) or cancer (61%)(× 2(2) = 8.00, *p* = .01). Those dying of cancer were more likely to have hospice involvement (30%) than those with chronic illness (12%) or dementia (5%) (× 2(2) = 19.57, *p* = .00) (Table 2).

**Table 2:** Sample demographics and characteristics (*n* = 286)

	Total sample n(%)	Cancer n(%)	Dementia n(%)	Cancer & Dementia n(%)	Chronic Disease n(%)	*p* ^b^
*Total Sample* ^*a*^	286 (100)	48 (17)	140 (49)	12 (4)	86 (30)	
Age						.20
Lowest to 80 years	64 (23)	23 (49)	22 (16)	3 (25)	16 (19)	
81 years to 91 years	126 (45)	14 (30)	75 (54)	7 (58)	30 (36)	
92 years and over	91 (32)	10 (21)	41 (30)	2 (17)	38 (45)	
Gender						
Male	104 (41)	25 (58)	52 (40)	3 (33)	24 (32)	.02
Female	152 (59)	18 (42)	77 (60)	6 (67)	51 (68)	
Ethnicity						.20
NZ European	232 (86)	39 (89)	115 (85)	7 (58)	71 (89)	
Maori	6 (2)	1 (2)	1 (1)	2 (17)	2 (3)	
Pacific Islander	1 (0)	0 (0)	1 (1)	0 (0)	0 (0)	
Asian	10 (4)	0 (0)	9 (7)	1 (8)	0 (0)	
Other	22 (8)	4 (9)	9 (7)	2 (9)	7 (9)	
Level of care at time of death						*.12*
Rest Home (low level)	28 (10)	2 (4)	11 (8)	2 (17)	13 (16)	
Private Hospital (high level)	217 (77)	46 (96)	93 (67)	(67)	70 (83)	
^c^Secure (dementia)	18 (6)	0 (0)	16 (12)	2 (17)	0 (0)	
^d^Psychogeriatric	18 (6)	0 (0)	18 (13)	0 (0)	0 (0)	
Other	2 (1)	0 (0)	1 (1)	0 (0)	1 (1)	
Resident able to express wishes about medical decisions						*.00*
Yes	114 (42)	32 (68)	34 (26)	3 (25)	45 (55)	
No	160 (58)	15 (32)	99 (74)	9 (75)	37 (45)	
End of life pathway/guideline used						*.81*
Yes	52 (19)	8 (17)	28 (21)	0 (0)	16 (20)	
No	218 (81)	39 (83)	103 (79)	11 (100)	65 (80)	
Local hospice involved in the last days of life						*.00*
Yes	31 (11)	14 (30)	7 (5)	0 (0)	10 (12)	
No	241 (89)	33 (70)	123 (95)	12 (100)	73 (88)	
Family members/friends involved in resident’s care during the last month of life						*.42*
Yes	243 (87)	43 (90)	120 (89)	10 (83)	70 (83)	
No	36 (13)	5 (10)	15 (11)	2 (17)	14 (17)	
CPR						*.51*
Yes	11 (4)	3 (7)	4 (3)	1 (9)	3 (4)	
No	249 (96)	39 (93)	123 (97)	10 (91)	77 (96)	
EPOA (Designated proxy decision maker)						*.00*
No	36 (13)	14 (33)	6 (4)	4 (40)	12 (15)	
Yes	233 (87)	28 (67)	129 (96)	6 (60)	70 (85)	
Death Expectation						*.10*
Faster than Expected	195 (73)	42 (88)	89 (69)	7 (64)	57 (72)	
Neither expected or unexpected	56 (21)	6 (13)	32 (25)	2 (18)	16 (20)	
Unexpected	17 (6)	0 (0)	9 (7)	2 (18)	6 (8)	
Sentinel Events Last Month of Life (multiple response)						
Pneumonia (yes/no)	70 (29)	7 (15)	40 (36)	1 (10)	22 (31)	.11
Febrile episode (except pneumonia) (yes/no)	28 (12)	4 (8)	15 (13)	0 (0)	9 (13)	.83
Problems eating/drinking (yes/no)	115 (48)	18 (38)	61 (55)	5 (50)	31 (44)	.43
Hip fracture (yes/no)	11 (5)	0 (0)	6 (5)	2 (20)	3 (4)	.34
Gastrointestinal Bleed (yes/no)	8 (3)	1 (2)	2 (2)	1 (10)	4 (6)	.31
Stroke (yes/no)	29 (12)	2 (4)	16 (14)	0 (0)	11 (16)	.23
Cancer (yes/no)	58 (24)	48 (100)	0 (0)	10 (100)	0 (0)	.00
Other (yes/no)	60 (25)	7 (15)	28 (25)	0 (0)	25 (35)	.07

^a^Missing values: primary diagnosis, gender, ethnicity, level of care, ability to express wishes, end of life pathway used, hospice use, family/friends involved, CPR, EPOA, death expectation. Percentage values represent actual percent not valid percent

^b^Comparisons based on diagnoses of cancer, dementia or chronic condition only. ^c^24 hour care in a secure environment for those with dementia. ^d^24 hour care in a secure environment for those with severe behavioural or psychological conditions

### Symptoms last month of life

There was no statistical difference (*p* > .05) in symptom management as assessed by the SM-EOLD total score across diagnostic categories in the last month of life (Table 3). However, there was a significant effect of diagnosis (cancer/dementia/chronic condition) on SM-EOLD subscale ratings of physical symptoms F (2, 247) = 5.05, *p* = .007.

**Table 3:** M-EOLD range during last month of life 0 (worst) to 5 (best) mean (SD) by diagnosis (*n* = 264)

	Mean Score	Range	Cancer *n* = 41	Dementia *n* = 114	Cancer + Dementia *n* = 8	Chronic Illness *n* = 74
Pain		0–5	.8(.8)	.6 (1.0)	.5(.7)	1.0 (1.4)
Shortness of breath		0–5	3.7 (1.7)	3.3 (1.9)	3.6 (1.9)	3.4 (1.8)
Skin breakdown		0–5	4.2 (1.3)	3.6 (1.8)	3.5 (2.1)	3.9 (1.6)
Calm		0–5	2.5 (1.7)	2.6 (1.9)	3.0 (2.2)	2.6 (1.8)
Depression		0–5	3.0 (1.8)	2.7 (1.8)	2.5 (2.2)	2.9 (1.8)
Fear		0–5	4.1 (1.5)	3.3 (1.8)	3.0 (2.2)	3.7 (1.6)
Anxiety		0–5	1.6 (1.6)	2.5 (1.8)	3.1 (2.3)	2.3 (1.9)
Agitation		0–5	2.9 (1.9)	3.4 (1.9)	3.6 (2.2)	3.1 (1.9)
Resistive to care		0–5	3.7 (1.7)	4.0 (1.5)	3.6 (2.2)	3.7 (1.8)
Total	26.7	0–45	26.9 (8.6)	26.5 (8.2)	26.3 (11.8)	26.9 (8.6)
Subscale Physical Symptoms	8.05	0–15	8.8 (2.7)	7.7 (3.1)	7.6 (3.0)	8.3 (3.3)
Subscale Psychological Symptoms	18.40	0–30	18.0 (5.9)	18.7 (6.3)	18.7 (9.4)	18.5 (6.5)

Planned contrast analyses between diagnosis groups revealed significantly fewer physical symptoms between those with cancer and those with dementia or chronic illness, t (247) = 3.14, *p* = .002, and between those with cancer and those with dementia t (247) = − 2.93, *p* = .004, but no significant differences between those with dementia and those with chronic illness t (247) = − 1.15, *p* = .24. There was also a significant effect of diagnosis on SM-EOLD ratings of anxiety, F (2, 251) = 4.61, *p* = .01. Planned contrast analyses revealed significant differences in anxiety for those with cancer (lower anxiety) and those with dementia or chronic disease, t (251) = 2.68, *p* = .008, and between those with cancer and those with dementia t (251) = − 3.03, *p* = .003. There were no significant differences in anxiety between those dementia or chronic illness t (251) = − 1.96,*p* = .05.

### Symptoms the last week of life

RNs rated symptoms in the last week of life using the Comfort Assessment at End-of-Life in Dementia scale (CAD-EOLD). Overall, there was no statistical difference (*p* > .05) in the end of life physical or emotional symptoms across diagnostic categories in the last week of life (Table 4). However, there was a significant effect of diagnosis (cancer/dementia/chronic condition) on CAD-EOLD physical distress subscale ratings F (2, 241) = 4.63, *p* = .01. Planned contrast analyses between diagnosis groups revealed no significant differences in physical distress between those with cancer and other diagnoses (dementia, chronic disease), t (241) = − 1.13, *p* = .26. There were significant differences in physical distress between those with dementia and cancer and those with chronic illness (more distress) t (241) = − 2.68,*p* = .008, t (230) = 2.59, *p* = .01 respectively. There was also a significant effect of diagnosis on CAD-EOLD ratings of pain, F (2, 240) = 4.92, *p* = .008. Planned contrast analyses revealed no significant differences in pain between those with cancer and those with dementia or chronic illness, t (240) = − 1.76, *p* = .07, but there were significant differences in pain between those with chronic illness or cancer and dementia (more pain) t (240),-2.37, *p* = .018, t (267), 2.67, *p* = .008. Finally, there was a significant effect of diagnosis for anxiety F (2, 237) = 3.55, *p* = .03. Planned contrast analyses demonstrated no significant differences in anxiety between those with cancer and those with dementia or chronic illness, t (237) = .43, *p* = .66, or between those with cancer and dementia t (237) = 1.53, *p* = .12. There were significant differences in anxiety between those with dementia and those with other chronic illness (more anxiety) t (237),2.57, *p* = .01. There were no other significant differences in CAD-EOLD items based on diagnosis (*p* > .05).

**Table 4:** CAD-EOLD range during the last week of life: 1 (worst) to 3 (best) mean (SD) (*n* = 221)

	Mean Score	*Range*	*Cancer* *n = 40*	*Dementia* *n = 104*	*Cancer + Dementia* *n = 11*	*Chronic Disease* *n = 65*
Discomfort	2.1 (.6)	1–3	2.0(.5)	2.3(.6)	2.0(.7)	2.0(.6)
Pain	2.24(.6)	1–3	2.0(.6)	2.3(.6)	2.0(.7)	2.1(.6)
Restlessness	2.2 (.7)	1–3	2.1(.7)	2.3(.7)	2.0(.8)	2.3(.7)
Shortness of breath	2.3 (.7)	1–3	2.2(.7)	2.4(.6)	2.3(.9)	2.2(.7)
Choking	2.8 (.4)	1–3	2.8(.3)	2.7(.5)	2.9(.3)	2.8(.4)
Gurgling	2.6 (.6)	1–3	2.6(.5)	2.5(.6)	2.9(.3)	2.6(.6)
Difficulty swallowing	2.2 (.7)	1–3	2.2(.7)	2.1(.7)	2.1(.8)	2.2(.7)
Fear	2.5 (.6)	1–3	2.4(.7)	2.6(.5)	2.5(.6)	2.5(.6)
Anxiety	2.2 (.7)	1–3	2.2(.7)	2.4(.6)	2.1(.8)	2.0(.7)
Crying	2.8 (.4)	1–3	2.7(.6)	2.8(.3)	2.9(.3)	2.7(.6)
Moaning	2.4 (.6)	1–3	2.4(.7)	2.5(.6)	2.2(.6)	2.4(.7)
Serenity	2.1(.7)	1–3	2.3(.6)	2.0(.8)	2.0(.7)	2.0(.7)
Peace	2.3(.6)	1–3	2.5(.6)	2.4(.7)	2.3(.6)	2.3(.6)
Calm	2.4(.6)	1–3	2.4(.6)	2.4(.6)	2.3(.6)	2.3(.6)
Total	33.7 (5.2)	14–42	33.3 (5.1)	34.4 (5.2)	33.1 (5.6)	33.0 (5.1)
*Subscale Physical Distress*	9.0 (2.1)	4–12	8.5 (2.0)	9.4 (2.0)	8.5 (2.7)	8.6 (1.9)
*Subscale Dying Symptoms*	9.9 (1.7)	4–12	10.0 (1.4)	9.9 (1.8)	10.3 (1.7)	9.9 (1.8)
*Subscale Emotional Distress*	10.15 (1.95)	4–12	9.7 (2.2)	10.5 (1.7)	9.9 (1.8)	9.7 (2.1)
*Subscale Wellbeing*	6.93 (1.92)	3–9	7.3 (1.8)	6.9 (1.9)	6.7 (1.7)	6.8 (1.8)

MANOVA was used to assess whether those with hospice involvement had higher levels of comfort in the last week of life (CAD-EOLD subscale scores) and whether there was an interaction between diagnosis and hospice involvement [The assumptions of independence of observations and homogeneity of variance/covariance were checked and met]. The interaction effect was not significant Wilk’s Λ = .96, F (8,392) = .95, *p* = .47. The main effects for hospice involvement Wilk’s Λ = .9, F (4,195) = 2.38, *p* = .05 and for diagnosis Wilk’s Λ = .96, F (8,390) = 1.22, *p* = .28 were also not significant.

## Discussion

This study presents a new perspective on end of life experiences in LTC settings using a comparison of illness trajectory by disease category. One of the main aims of the research was to provide a rare replication of other nationwide studies of end of life in LTC [10, 16]. New Zealand was found in this study to rank highly in overall quality of LTC end of life care in comparison to other similar studies [10, 21]. Additionally, while differences between cancer and dementia have been examined in previous research, little research has explored differences between persons with a diagnosis of dementia and those with a diagnosis of a chronic condition [7, 13, 22]. This study replicated the methods of a nationwide Belgian study of quality of dying for people with dementia in LTC so that a meaningful comparison could be made [10]. In the Belgian study, the mean EOLD-CAD score for people with dementia in the last week of life was 30.0 compared with this New Zealand study where the mean CAD-EOLD score was 33.8 overall and 34.4 for people with dementia (higher scores indicate better quality) [10]. The recent PACE study examined quality of death in LTC across six European countries provides a multi-country comparison. This study found the mean CAD-EOLD score for symptoms in the last week of life ranged from 29.9 to 33.9 across the six participating countries [21].

Although this study reveals a relatively good quality of end of life care in New Zealand LTC facilities, other studies indicate that there is room to improve. For instance, in a small prospective study, people dying of dementia were deemed to have a low symptom burden at death with mean scores of 35.1 [23]. The cluster-randomised CAREful intervention study examined end of life care for older people dying in an acute care geriatric ward and found the CAD-EOLD mean score post intervention was 34.6 [24].

The factors impacting the quality of LTC end of life care in New Zealand compared to other countries are unclear. A contributing factor could be that New Zealand has one of the highest proportion of deaths in LTC (38% of all deaths) compared to other resource rich countries and therefore staff may have more experience in end of life care overall [1]. In New Zealand there have also been national initiatives to implement end of life guidelines in LTC. This may have affected end of life quality, particularly because in this study guidelines were used in approximately one in five deaths, regardless of diagnosis [25, 26].

All residents, regardless of primary diagnosis, had similar physical and emotional characteristics in their last week of life. However, in this study those with dementia or a chronic condition had significantly greater physical symptoms in the last month of life compared to those with cancer, although those with chronic illness had more anxiety. The needs of residents with end-stage dementia have been found to be similar to those with cancer. These include shortness of breath, skin breakdown, infections and constipation[8]. Nonetheless, this study found the above symptoms can be experienced for a much longer period of time for those with dementia (median length of stay 15 months) and chronic illness (median length of stay 17 months) compared to those with cancer (median length of stay 1 month). People living in LTC with advanced physical and cognitive frailty require complex and integrated geriatric and palliative care in the months and possibly years prior to death [5]. This study supports Hockley’s assertion that a palliative care model developed for cancer should not be imposed onto frail older people dying in LTC facilities [27].

In this study those diagnosed with cancer were twice as likely to have community hospice involvement. Although in recent years more people with non-malignant illness are being cared for by specialist palliative care providers, the majority of hospice care continues to be provided for those with cancer [28]. This study found no difference in comfort in the last week of life regardless of community hospice involvement, although the numbers were relatively small.

In this study none of the sample with a primary diagnosis of cancer were considered to have died unexpectedly, compared to 7% of those with dementia and 8% of those with chronic illness. This finding relates to one of the most difficult aspects of palliative care for older people dying of non-malignant disease, which is prognostication of death [3]. This issue is not unique to New Zealand. Several studies have attempted to develop tools to predict end of life in order to ensure that appropriate palliative care is provided [9]. In a recent study, the provider question “would you be surprised if this patient died in the next 12 months” had a relatively poor ability to predict death for those without cancer [29]. Other studies have demonstrated the characteristics of frailty such as cognitive impairment and functional disability may be a useful way to identify changing care needs and predict time to death [4].

As with all studies, this research has strengths and limitations. A strength is that the sample was representative of all facilities across New Zealand and therefore provides a rare robust overview of deaths in LTC across an entire country [10]. As with all palliative care research, it is very difficult to collect data from the people who are dying or to prognosticate potential death for those in LTC with non-malignant conditions [30]. For large scale epidemiological studies, post-death proxy data collection has been found to be the most accurate and feasible method. Although recall bias cannot excluded, attempts were made to mitigate this by identification of the staff most involved in the care at the end of life and by follow up data collection within 2 weeks of death. For this study, the tools used were developed for those with dementia but have been assessed as appropriate to determine quality of dying for all LTC residents and were used in a recent large multi-national European study [18–21]. A further limitation is that there were no independent observations and quality of end of life was evaluated by staff only, although in another study, staff have been found to provide a more valid assessment of symptoms than families [31]. It is questionable whether physical and emotional symptoms can be accurately assessed by others, but this is an accepted and ethical means of evaluating symptoms for those in the last days of life [10, 21, 30]. Finally, the small number of cases of those with diagnoses of both cancer and dementia (*n* = 12) prevented comparisons of this group with other diagnostic categories. Future research with larger samples could usefully elaborate the impact of dual diagnoses on resident end of life in LTC.

This study’s finding of a high symptom burden in the last weeks of life for those dying in LTC, particularly for those with dementia and chronic illness has implications for the care in LTC. The resources and staffing available in LTC do not always reflect the needs of residents placing stress on the staff and impacting overall quality of care [32]. It is interesting that in this study, traditional community hospice involvement did not affect quality of dying. Innovative models of care, including reviews of effective staffing levels, are needed in order to provide high quality end of life care. One example is the SHARE intervention that proactively integrates hospice support into LTC. This includes on-going monitoring of the overall palliative care needs of all residents and staff debriefing. This model has demonstrated significant decreases in staff depression, burn-out and greater confidence in providing end of life care [33]. With significant increases in the number of deaths of the oldest-old predicted, it is crucial for specialist palliative care providers to understand gerontology and frailty, as much as it is for those caring for the frailest of older people to understand palliative care philosophy and practice. This points to the need for dual training in gerontology and palliative care for nursing and medical staff across acute, hospice and LTC settings [34].

## Conclusion

This study found that New Zealand ranks highly in overall LTC end of life care in comparison to other countries. We also found that symptom management needs in the last week of life do not vary by diagnosis overall, although sub-group analysis found residents with dementia and chronic illness experience higher physical distress over a longer period of time before death than residents with cancer. Residents with advanced physical and cognitive frailty often require long-term care for complex geriatric issues which need to be integrated with palliative care principles in the months and possibly years before they die. It is essential that those working in LTC facilities recognise palliative care philosophy and practice as an integral part of their work and that the model of care acknowledges the demands associated with LTC end of life care. It is also crucial that specialist palliative care providers work collaboratively with, and become more skilled in gerontology and complex geriatric syndromes. Now, and into the future, the oldest old will make up the majority of all deaths, and a significant proportion of people will die in LTC. It is therefore essential that gerontology and palliative care approaches are integrated to assure high quality end of life care in LTC settings.

## Abbreviations

CAD-EOLD: Comfort Assessment at End of life in Dementia
CPR: Cardiopulmonary resuscitation
GPs: General medical practitioners
LTC: Long term care
NHI: National health identification number
NPs: Nurse practitioners
RNs: Registered nurses
SM-EOLD: Symptom Management at End of life in Dementia
